# Single fraction radiosurgery/stereotactic body radiation therapy (SBRT) for spine metastasis: A dosimetric comparison of multiple delivery platforms

**DOI:** 10.1002/acm2.12022

**Published:** 2016-12-29

**Authors:** Adrian Nalichowski, Isaac Kaufman, John Gallo, Todd Bossenberger, Tim Solberg, Ezequiel Ramirez, Yulong Yan, Julie Fredrick, Tewfik Bichay, Alan Mayville, Jay Burmeister

**Affiliations:** ^1^ Department of Oncology Karmanos Cancer Institute Detroit MI USA; ^2^ Department of Oncology Wayne State University School of Medicine Detroit MI USA; ^3^ Department of Radiation Oncology University of Texas Southwestern Medical Center Dallas TX USA; ^4^ Department of Radiation Oncology Huron Valley Sinai Hospital Commerce MI USA; ^5^ Lacks Cancer Center Radiation Oncology Saint Mary's Health Care Grand Rapids MI USA

**Keywords:** RTOG 0631, SBRT, spine metastasis, SRS

## Abstract

There are numerous commercial radiotherapy systems capable of delivering single fraction spine radiosurgery/SBRT. We aim to compare the capabilities of several of these systems to deliver this treatment when following standardized criteria from a national protocol. Four distinct target lesions representing various case presentations of spine metastases were contoured in both the thoracic and lumbar spine of an anthropomorphic SBRT phantom. Single fraction radiosurgery/SBRT plans were designed for each target with each of our treatment platforms. Plans were prescribed to 16 Gy in one fraction to cover 90% of the target volume using normal tissue and target constraints from RTOG 0631. We analyzed these plans with priority on the dose to 10% of the partial spinal cord and dose to 0.03 cc of the spinal cord. Each system was able to maintain 90% target coverage while meeting all the constraints of RTOG 0631. On average, CyberKnife was able to achieve the lowest spinal cord doses overall and also generated the sharpest dose falloff as indicated by the Paddick gradient index. Treatment times varied widely depending on the modality utilized. On average, treatment can be delivered faster with Flattening Filter Free RapidArc and Tomotherapy, compared to Vero and Cyberknife. While all systems analyzed were able to meet the dose constraints of RTOG 0631, unique characteristics of individual treatment modalities may guide modality selection. Specifically, certain modalities performed better than the others for specific target shapes and locations, and delivery time varied significantly among the different modalities. These findings could provide guidance in determining which of the available modalities would be preferable for the treatment of spine metastases based on individualized treatment goals.

## 1 Introduction

Spinal metastases are a common oncologic occurrence that can have a major impact on the cancer patient's quality of life and functional status. It is well known that radiation therapy is an excellent palliative treatment for spine metastases. Currently, accepted radiation techniques include a variety of fractionated regimens as well as single fraction treatment, which has historically been delivered at a dose of 8 Gy. Multiple studies have shown these techniques to result in a pain response of approximately 60%.^1^, ^2^ More recent data support the use of stereotactic body radiation therapy (SBRT) or radiosurgery for spinal metastases with fewer fractions delivered and greater, more durable responses. Gerszten et al. reported that 86% of patients experienced long‐term pain improvement and excellent local control utilizing SBRT.^3^ In current practice, an increasing percentage of patients with spine metastases can experience long‐term survival. As systemic therapy continues to improve, it becomes even more important to produce durable pain palliation and local control.^4^

SBRT is commonly defined as a treatment that couples a high degree of anatomic targeting accuracy and reproducibility with very high doses of extremely precise, externally generated, ionizing radiation delivered in five or fewer fractions to an extracranial target. Treatment consisting of one fraction only is referred to as radiosurgery. The use of radiosurgery/SBRT has increased significantly over the last several years. A recent survey of radiation oncologists practicing in the United States reported that 63.9% use SBRT for selected patients, the most common treatment locations including lung, spine, and liver.^5^ As utilization of this technique increases, so have the number of platforms designed to deliver such treatment. At our own institution, we have multiple treatment planning and delivery systems used for highly conformal SBRT treatments, but no set guidelines for choosing between them.

Previous reports on modality selection for SRS/SBRT have been published for intracranial sites and were either limited to two platforms^6^ or compared based on technical specifications.^7^ Within this study, we attempt to determine whether there are significant differences in planning and delivery capabilities across these platforms within the context of the current RTOG 0631 radiosurgery/SBRT spine trial. Therefore, we designed sample spine metastasis cases within a phantom model and generated radiosurgery treatment plans for five different planning and delivery systems. We hypothesized that, while each modality would be able to meet the constraints of RTOG 0631, there would be differences in dose to critical normal tissue, treatment time, and dose fall off that may assist in the choice of delivery system based on characteristics of the individual case and target shape/volume.

## 2 Methods and materials

**Figure 1:**
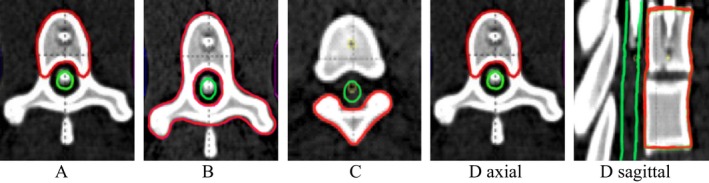
Axial representations of target volumes (Red) and spinal cord (Green) along with sagittal image of target “D” to illustrate its extent across two vertebral levels.

Radiotherapy treatment simulation was performed on an anthropomorphic thorax/abdomen phantom (Integrated Medical Technologies, Troy, NY, USA) using a 40‐slice CT scanner with 1.5 mm slice thickness. Sample spine radiosurgery plans were created using idealized target volumes created on the phantom. Target volumes were designed using the Eclipse (Varian Medical Systems, Palo Alto, CA, USA) treatment planning system in accordance with parameters in the International Spine Radiosurgery Consortium Consensus Guidelines for Target Volume Definition in Spinal Stereotactic Radiosurgery^8^ and the recommendations of RTOG 0631. Four distinct target volumes representing typical case presentations of spine metastases were contoured in both the thoracic and lumbar spine of our anthropomorphic phantom. Target volume “A” included a single vertebral body, target volume “B” included all elements of a single vertebral level completely encircling the spinal cord, target volume “C” included only the spinous process of a single vertebral level, and target volume “D” included two consecutive vertebral bodies (Fig. 1). The spinal cord was contoured as a structure approximately 7 mm in diameter, contained centrally within the bony limits of the spinal canal. The contoured cord was designed to reflect the average cord size of our previous ten radiosurgery spine patients who were planned using fusion of MRI or CT myelogram as well as measurements reported in the literature.^9^ To ensure comparability, each contour and image set was communicated unaltered from Eclipse to each of the other treatment planning systems through DICOM‐RT.

Dose objectives and constraints were designed to meet those required for RTOG 0631 and the target was prescribed 16 Gy in a single fraction. Briefly, planning requirements included the following: at least 90% of the target volume receives the prescribed radiosurgery dose; hotspots outside the target were limited to 105% within 1 cm of the target volume and 110% anywhere outside the target. Spinal cord constraints included 10 Gy to 10% of the partial spinal cord defined as 5–6 mm above and below the target, the total volume of spinal cord receiving 10 Gy was restricted to below 0.35 cc, and the absolute maximum dose to the spinal cord was restricted to 14 Gy to a volume of no more than 0.03 cc. Additional OAR constraints included cauda equina volume of < 0.03 cc receiving 16 Gy, and < 5 cc receiving 14 Gy. The total lung was limited to a volume of less than 1000 cc receiving 7.4 Gy. A point dose of 110% of the prescribed dose was allowed outside the target volume as long as it was less than 0.03 cc, which is an acceptable variation per the protocol.

With these objectives, single fraction radiosurgery plans were designed for each target to be delivered with CyberKnife (CK) with Iris collimator (Accuray, Sunnyvale, CA, USA), Tomotherapy with TomoEDGE™ dynamic jaw (Tomo) (Accuray Sunnyvale, CA, USA), Vero (BrainLAB, Feldkirchen, Germany and Mitsubishi Heavy Industries, Tokyo, Japan), and Varian TrueBeam (Varian Medical Systems, Palo Alto, CA, USA), the latter utilizing RapidArc (RA) in standard and flattening filter free (FFF) modes. The plans for each system were designed by experienced dosimetrists and physicists responsible for planning clinical cases on these systems. Within the constraints listed, the planner was asked to design the best possible plan with priority on the spinal cord constraints. The planners were blinded to the planning techniques and results of other modalities so as to not influence their results. Each institution chose planning (dose calculation algorithm, grid size, etc.) and machine parameters (number of fields, pitch, gantry angles, etc.) according to their clinical practice.

For the Varian TrueBeam RapidArc with and without flattening filter plans, the Eclipse (v. 10.0.39) treatment planning system was used to create two complete arcs. Dose calculation was performed using the Analytical Anisotropic Algorithm (AAA) using a 2.5 mm dose grid. The iPlan (v. 4.1.2) treatment planning system was used to create the Vero treatment plans, utilizing a 2 mm dose grid. Thirteen coplanar IMRT beams were uniformly distributed through 360 degrees, and the Monte Carlo dose algorithm was used. For Cyberknife, the MultiPlan (V 5.1) treatment planning system was used with the Monte Carlo Algorithm with high resolution and a 1.0 mm × 1.0 mm × 1.5 mm grid for dose calculation. For the TomoEdge plans, the Tomotherapy VoLO (v. 5.0.0.0) treatment planning system was used with a 2 mm grid size used for dose calculations.

We analyzed these plans with priority on the dose to 10% of the partial spinal cord and dose to 0.03 cc of the spinal cord. The Paddick dose gradient index (PGI), defined as the ratio of the volume encompassed by half the prescription dose to the volume encompassed by the prescription dose, was used as a measure of the steepness of the dose gradient around the target.^10^ Once we confirmed that each system was able to meet all of the target goals of the protocol, we compared these two cord metrics along with their ability to limit the dose to other surrounding tissues using the PGI.

## 3 Results

**Figure 2:**
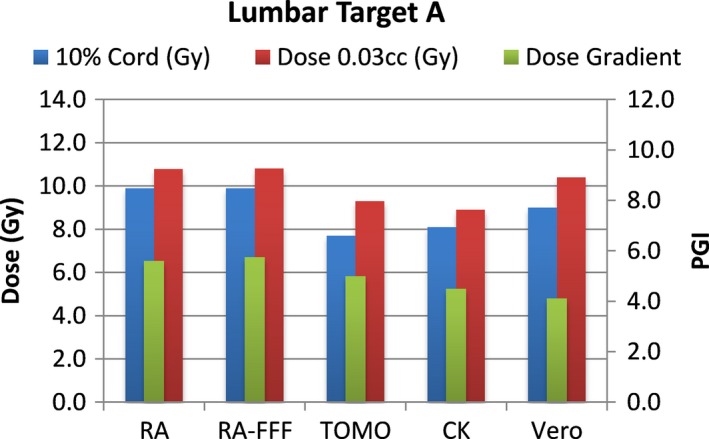
Dose to 10% of partial cord, maximum dose to 0.03 cc and PGI.

**Figure 3:**
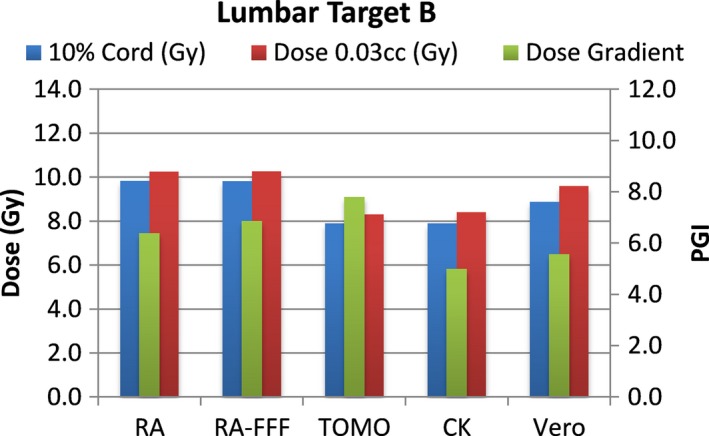
Dose to 10% of partial cord, maximum dose to 0.03 cc and PGI.

**Figure 4:**
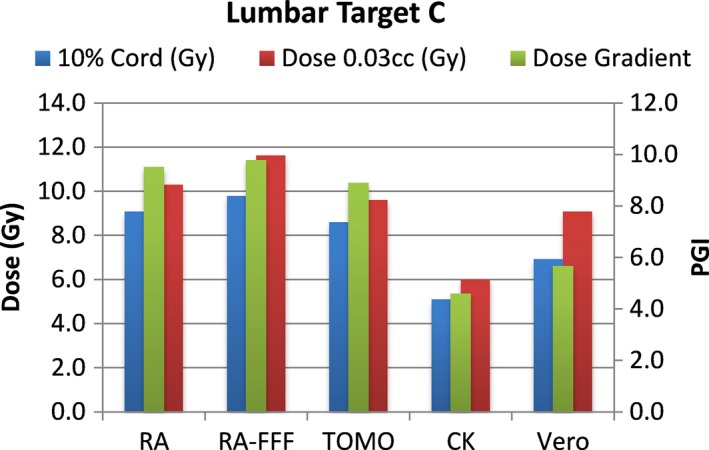
Dose to 10% of partial cord, maximum dose to 0.03 cc and PGI.

**Figure 5:**
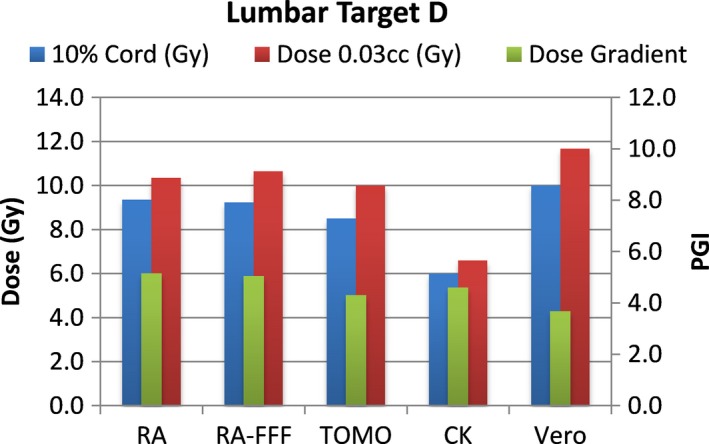
Dose to 10% of partial cord, maximum dose to 0.03 cc and PGI.

**Figure 6:**
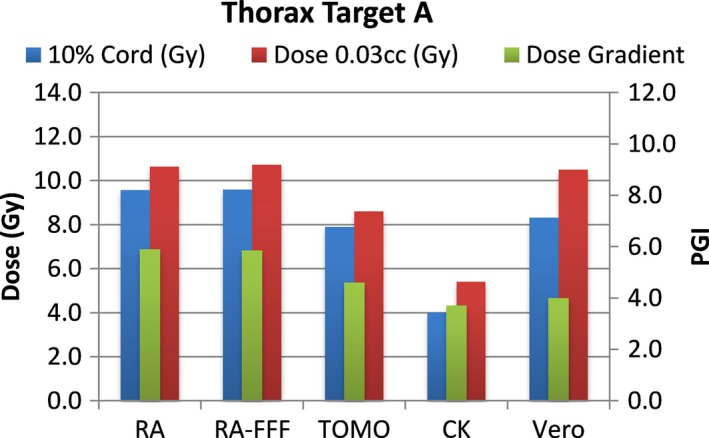
Dose to 10% of partial cord, maximum dose to 0.03 cc and PGI.

**Figure 7:**
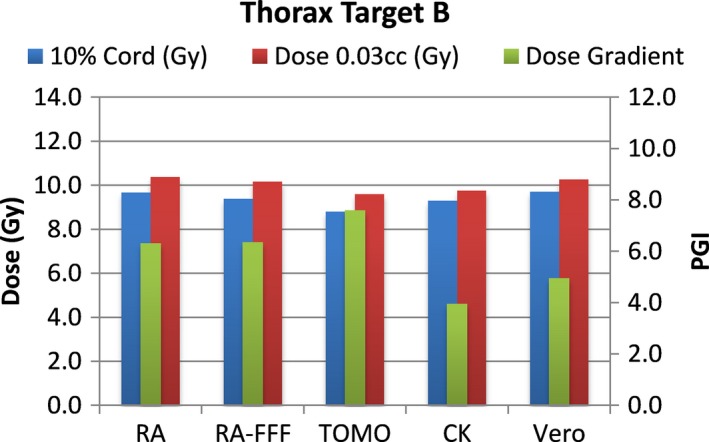
Dose to 10% of partial cord, maximum dose to 0.03 cc and PGI.

**Figure 8:**
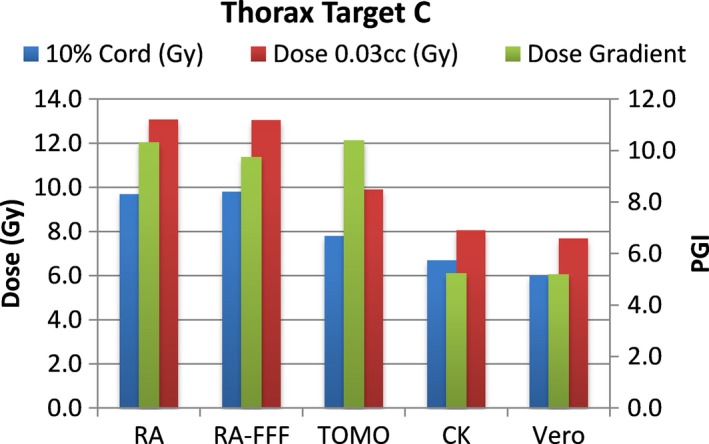
Dose to 10% of partial cord, maximum dose to 0.03 cc and PGI.

**Figure 9:**
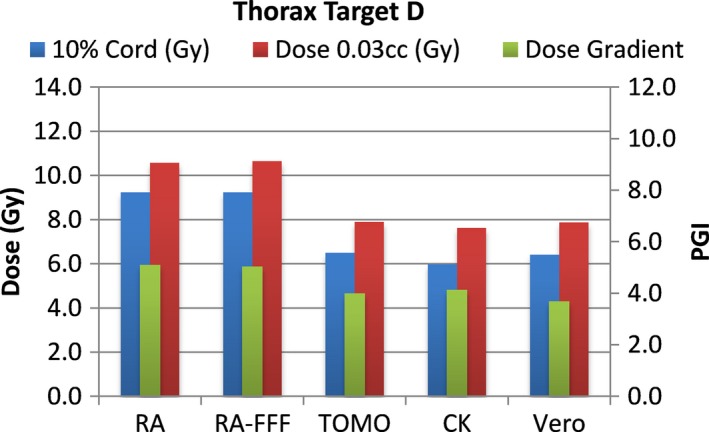
Dose to 10% of partial cord, maximum dose to 0.03 cc and PGI.

**Figure 10:**
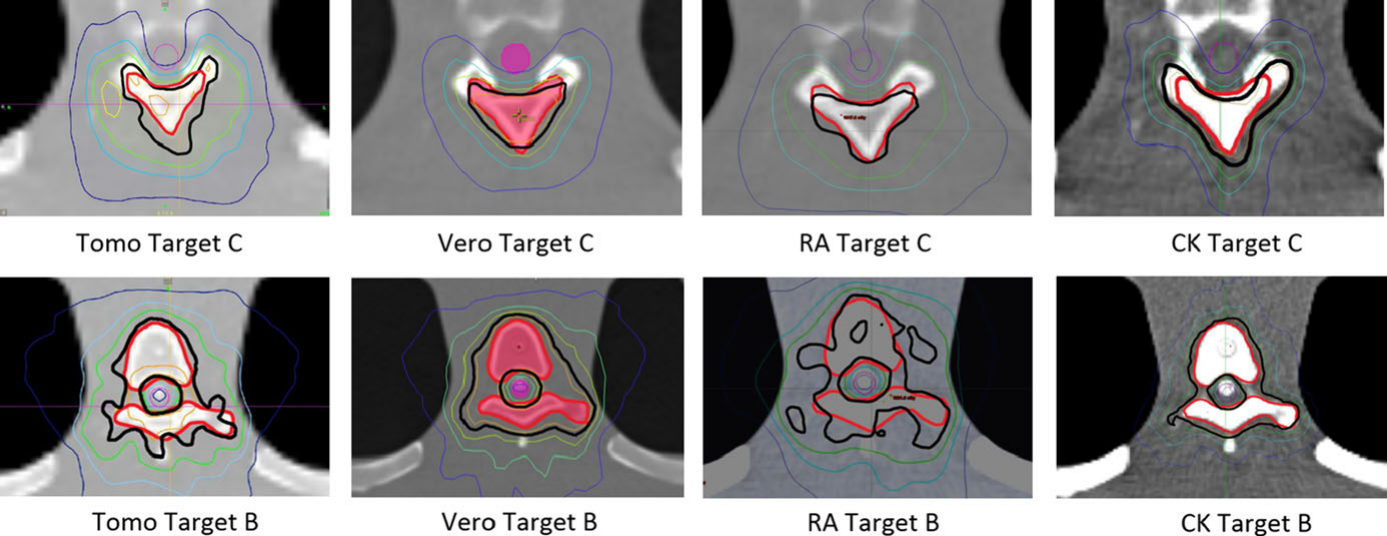
Isodose distributions for Targets B and C (Red) and their relationship with the spinal cord (Purple). Isodose lines are as follows: 16.8 Gy Orange; 16.0 Gy Black; 14.4 Gy Green; 12.0 Gy Light Blue; 8.0 Gy Dark Blue.

A total of 40 plans were generated for the cases listed above (eight for each platform—one for each lumbar and thoracic target). Each system was able to generate plans—delivering the prescription dose to 90% of the target volume while meeting all the constraints of RTOG 0631. RA and Tomo achieved the most homogeneous dose distribution within the target. D95 was on average 99.5% and 99.2% of the prescription dose for RA and Tomo, respectively. D95 results for CK and Vero were on average 93.0% and 95.2%, respectively. Target volume C, which was the smallest of the targets, showed the most variability between modalities. For example, the maximum cord dose to 0.03 cc was 13.1 Gy for RA‐FFF versus only 7.7 Gy for Vero. Both Vero and CK had lower cord doses and sharper dose falloff than the other modalities for target C. On average, as displayed in Figs. 2, 3, 4, 5, 6, 7, 8, 9, CK was able to achieve the lowest cord doses overall and also generated the sharpest dose falloff as indicated by the Paddick gradient index. Figure 10 provides a visual representation of the dose distribution created by each modality for the two most complicated cases, B and C. Treatment times varied widely depending on the modality utilized. On average, treatment can be delivered faster with RA‐FFF and Tomo, compared to RA, Vero and CK (Table 1). It should be noted that we are reporting treatment times for Tomotherapy Edge with dynamic jaws. Treatment times for Tomotherapy with static jaws were 3 to 5 times longer than with Tomo Edge depending on the target.

**Table 1:** Average treatment times across all targets for each modality

Modality	Average (min)	Range (min)
RA	9.5	7.2–11.2
RA‐FFF	4.4	3.5–4.9
Tomo	6.0	5.0–6.8
CK	58.1	32.0–85.0
Vero	19.1	15.0–24.5

## 4 Discussion

With the rapid innovation that is characteristic of the field of radiation oncology, it is important to ensure that adoption of new technology is done with a priority on safety. The delivery of high doses per fraction with radiosurgery/SBRT decreases our margin for error compared with traditional fractionated radiation. Especially in spine radiosurgery/SBRT cases with tumors adjacent to the spinal cord, it is critically important to minimize dose to normal structures while at the same time maintaining the ability to deliver adequate dose to the target. In our study, we have shown the dosimetric results of a representative set of cases planned with multiple delivery platforms. These do not by any means encompass the wide variety of cases seen in clinical practice, nor do the 40 plans generated in our study represent the capabilities of other planning teams. In addition, we recognize that small inaccuracies in patient setup and variability in actual treatment delivery can have serious and significant consequences that could far exceed the differences between modalities that are presented here. Indeed, evaluation of the relative ability of each of these systems to accurately deliver these treatment plans is critical in determining whether there is any real advantage to one over another.

In an associated quality assurance analysis study,^11^ both ion chamber and film were used to measure delivered dose for all plans on each modality presented here. The results of these measurements were exceptional, specifically that all ion chamber measurements were within 3.3% of the dose predicted by the respective treatment planning system and all modalities yielded film gamma pass rates better than 96% at 2%/2mm. Finally, while we identified situations in which some systems provide a dosimetric advantage in treatment plan characteristics for a particular SBRT spine treatment, it is not clear if these differences would translate into a clinical advantage.

We believe that the ability of CK to achieve overall superior dosimetric results comes from use of the smallest aperture and a greater number of possible beam orientations. The CK was the only modality in this study that used non‐coplanar beams. The difference between the Vero and TrueBeam results could be attributed to the number of beams since 13 coplanar beams were used for the Vero plans and only two arcs were used for the TrueBeam plans. Both machines have an MLC leaf width of 5mm. Also, Burghelea et al.^6^ showed that smaller aperture and non‐coplanar beams produce plans with better conformity and dose gradient for small targets. Both TrueBeam and Vero could benefit from using non‐coplanar beams. In addition, the use of the high definition MLC (HDMLC), which has 2.5mm leaf widths, could be expected to further improve dosimetric results for the TrueBeam plans.

The main objective of this study was to meet or exceed the dose constrains of RTOG 0631 protocol. Each institution was able to choose optimization and machine parameters according to their clinical practice. The results presented here may be influenced by the difference in dose grid size used by each platform. Other studies suggest that variation in dose calculation for grid sizes used in our study could be around 2–3%.^12^, ^13^

Depending on the goals of treatment, the order of importance of the treatment plan metrics we reported might vary. For example, in the case of a terminal patient with significant difficulty lying in the treatment position, the physician might decide that a short treatment time is more important than a sharp dose gradient or the potential risk of late neurologic complications. However, a patient with a better prognosis might tolerate the treatment well, and in that case lower doses to critical structures would justify a longer treatment time.

## 5 Conclusion

While all treatment modalities tested were able to create and very accurately deliver treatment plans meeting the dose constraints of RTOG 0631, we observed variations that may impact system selection based on individualized treatment goals. Certain modalities performed better than the others for specific target shapes and locations. Vero and CK excelled in treating small volume targets. CK had the sharpest dose falloff and achieved the lowest overall spinal cord doses at the expense of longest treatment time. Treatment delivery time was fastest for TB‐FFF and Tomo. These findings could provide guidance in the process of determining which of the available modalities would be preferable for the treatment of spine metastases.

## Conflict of Interest

None.
